# Fetal/Neonatal Alloimmune Thrombocytopenia: Pathogenesis, Diagnostics and Prevention

**DOI:** 10.1007/s00005-015-0371-9

**Published:** 2015-11-12

**Authors:** Ewa Brojer, Anne Husebekk, Marzena Dębska, Małgorzata Uhrynowska, Katarzyna Guz, Agnieszka Orzińska, Romuald Dębski, Krystyna Maślanka

**Affiliations:** Department of Immunohematology and Immunology of Transfusion Medicine, Institute of Hematology and Transfusion Medicine, Warsaw, Poland; Institute of Medical Biology, UiT The Arctic University of Norway, Tromsø, Norway; 2nd Department of Obstetrics and Gynecology, Medical Centre of Postgraduate Education, Warsaw, Poland

**Keywords:** Human platelet alloantigens, Pregnancy, Fetal/neonatal alloimmune thrombocytopenia, Therapeutic intervention

## Abstract

Fetal/neonatal alloimmune thrombocytopenia (FNAIT) is a relatively rare condition (1/1000–1/2000) that was granted orphan status by the European Medicines Agency in 2011. Clinical consequences of FNAIT, however, may be severe. A thrombocytopenic fetus or new-born is at risk of intracranial hemorrhage that may result in lifelong disability or death. Preventing such bleeding is thus vital and requires a solution. Anti-HPA1a antibodies are the most frequent cause of FNAIT in Caucasians. Its pathogenesis is similar to hemolytic disease of the newborn (HDN) due to anti-RhD antibodies, but is characterized by platelet destruction and is more often observed in the first pregnancy. In 75 % of these women, alloimmunization by HPA-1a antigens, however, occurs at delivery, which enables development of antibody-mediated immune suppression to prevent maternal immunization. As for HDN, the recurrence rate of FNAIT is high. For advancing diagnostic efforts and treatment, it is thereby crucial to understand the pathogenesis of FNAIT, including cellular immunity involvement. This review presents the current knowledge on FNAIT. Also described is a program for HPA-1a screening in identifying HPA-1a negative pregnant women at risk of immunization. This program is now performed at the Institute of Hematology and Transfusion Medicine in cooperation with the Department of Obstetrics and Gynecology of the Medical Centre of Postgraduate Education in Warsaw as well as the UiT The Arctic University of Norway.

## Introduction

Fetal/neonatal alloimmune thrombocytopenia (FNAIT), caused by maternal alloantibodies against fetal human platelet antigens (HPA) is a relatively rare condition (1/800–2000 live newborns) but its consequences may be severe (Kjeldsen-Kragh et al. 2007; Uhrynowska et al. 2000; Williamson et al. 1998). Over 80 % of FNAIT cases result from mother and fetus incompatibility to the human platelet antigen-1a (HPA-1a); the remaining ones arise, respectively, from antibodies against HPA-5b on GPIa (15 %) and those against other HPAs (5 %) (Peterson et al. 2013; Sachs 2013). A thrombocytopenic fetus or new-born is at risk of intracranial hemorrhage (ICH) that may result in lifelong disability or death. The disease may appear in the first pregnancy, but since there are no screening programs, FNAIT is currently diagnosed only after birth of a thrombocytopenic baby. Many FNAIT cases are not properly diagnosed or treated. It is, therefore, particularly important to identify those pregnancies at risk of fetal/neonatal thrombocytopenia caused by alloimmunization requiring treatment, together with developing new methods for noninvasive and effective treatment for preventing fetal bleeding; the latter being a key issue that needs to be resolved. This review aims to present the clinical characteristics of FNAIT and its pathogenesis, including factors that determine antibody generation and disease severity. We also discuss in summary current treatment methods, future developments as well as the perspectives of immunoprophylaxis. Furthermore, the legitimacy and perspectives of screening methods for eliminating FNAIT risk is presented in light of the current methods for preventing hemolytic disease of the newborn (HDN) due to red blood cell alloimmunization.

## Pathogenesis of FNAIT; Cellular and Humoral Immunity

Thrombocytopenia in the fetus or newborn is most commonly caused by viral or bacterial infections. However, it can also have an immunological etiology due to maternal auto- or alloantibodies. Autoantibodies are produced by patients with immune thrombocytopenic purpura (ITP) or systemic lupus erythematosus which bind to both maternal and fetal platelets. Maternal alloantibodies usually do not induce thrombocytopenia in the mother but do so to the fetus. Nevertheless, it should be stressed that alloantibodies can be also produced by mothers with ITP. The production of alloantibodies against a fetal platelet specific antigen, which is unknown to the maternal immune system, constitutes the basis of FNAIT pathogenesis.

Maternal IgG is actively transferred to the fetus via the neonatal Fc receptor (FcRn). IgA and IgM are not transferred because there are no specific receptors (Kumpel 2012; Kumpel and Manoussaka 2012; Leach et al. 1996). After crossing the placenta, maternal alloantibodies opsonize fetal platelets (PLTs) that are subsequently destroyed.

Alloantibody production by the maternal immune system is initiated when platelet antigens are presented by professional antigen presenting cells in maternal lymph nodes and spleen (Kumpel and Manoussaka 2012). This may result from fetomaternal hemorrhage, which can occur during delivery or miscarriage, as a result of platelet leakage into the maternal circulation. The women patients’ obstetric history is also important and must be considered carefully.

The alloimmunization by HPAs might be caused by very small (<500 nm) syncytiotrophoblast microparticles (STMP) released from syncytiotrophoblasts (ST) (Kumpel and Manoussaka 2012; Smith et al. 1977). The presence of glycoprotein GPIIIa which contains many HPA epitopes, including the most immunogenic one—HPA-1a, has been shown on STMP complexed with CD51 (αV integrin) (Kumpel et al. 2008). Platelet antigens are expressed in the fetus from gestational week 16 (Gruel et al. 1986). Trophoblasts, which are the interface between fetal and maternal tissue on one side and blood on the other, escape allorecognition because they do not express the classical HLA class I and II molecules, but, almost uniquely, express HLA-G which is a non-classical HLA class I molecule. This local immunoregulation leads to fetal alloantigen tolerance and prevents a killing of fetal and placental cells by maternal natural killer cells, cytotoxic T cells and macrophages. ST at the surface of chorionic villi, together with ST debris that shed into maternal blood, create a state of mild maternal systemic inflammation by enhancing innate immunity and prevents humoral immunity. This mode of immunization is presented in several reviews (Kumpel 2012; Kumpel and Manoussaka 2012; Warning et al. 2011).

The mechanisms that must be activated for producing platelet-reactive antibodies, has been carefully studied (Ahlen et al. 2009; Jackson et al. 2005; Kuwana et al. 2002; Maslanka et al. 1996; Wu et al. 1997). IgG antibody production requires interaction between T and B cells for the same antigen complex. T cell activated B cells differentiate into antibody producing plasma cells. In FNAIT, due to anti-HPA-1a, the platelet-reactive antibodies bind to an allogeneic epitope located on integrin β3 (defined by leucine at residue 33); a much less frequent alloform has a proline at this position and is defined as HPA-1b (Newman et al. 1989).

## Antigen Presentation

Not all women with the HPA-1bb genotype (phenotype HPA-1a negative) who give birth to an HPA-1ab (HPA-1a positive) child are immunized. Some may not be exposed to fetal antigens or the maternal genetic constitution is not suitable for the immune response. It is a well-accepted fact that the antigen must be presented to the maternal T cells mediated by certain HLA class II molecules. It was found that HPA-1a alloimmunization is initiated by binding of the HPA-1a peptides to HLA DRB3*01:01 molecules (Ahlen et al. 2009; Parry et al. 2007; Rayment et al. 2009; Wu et al. 1997). In the 1990s, Jack Gorski group (Maslanka et al. 1996; Wu et al. 1997) studied HPA-1a antigen recognition by T cells in the context of HLA DRB3*01:01. In the paper published by Maslanka et al. (1996) the authors describe a study in which they used T-cell receptor (TCR) spectratyping and PCR techniques with clonotypic primers to track T cells from women who delivered FNAIT babies. The cells were grown in a culture and stimulated with peptide containing the HPA-1a epitope. Molecular analysis of the responding T-cell repertoire identified two T cells that predominated in the culture stimulated with the alloantigen peptide. Detailed analysis revealed that two predominant clonotypes were involved. They shared a recognizable CDR3 motif (L-P-S/T) that was present in two different TCR Vβ families (Vβ5.3 and Vβ11). The presence of a CDR3 motif indicates specific selection for HPA-1a antigen recognition (Hedrick et al. 1988; Maslanka et al. 1996). T cells with TCR BV family were considered candidates for providing the HLA-DR restricted help for production of FNAIT-associated anti-platelet alloantibodies. However, further studies with peripheral blood mononuclear cells (PBMCs) from other mothers who had given birth to FNAIT babies did not confirm the presence of the same CDR3. This may suggest that a broader repertoire of HPA-1a specific T cells is involved in the cell mediated immune response to HPA-1a peptide. Studies by other authors demonstrated proliferative responses in peripheral blood mononuclear cells from HPA-1a-immunized women cultured with HPA-1a peptides (Jackson et al. 2005). This was supported by finding numerous different HPA-1a specific T-cell clones isolated from a HPA-1a immunized woman (Ahlen et al. 2009). PBMCs were cultured from an HPA-1a-alloimmunized woman in the presence of HPA-1a peptide, labeled with CFSE, and assayed for antigen-specific proliferation. Individual proliferating cells were isolated by fluorescence-activated cell sorting and expanded in culture. Antigen specificity and HLA restriction were determined by cytokine secretion (enzyme-linked immunospot) and proliferation assays. Several isolated CD3^+^CD4^+^ T-cell clones proliferated and secreted cytokines in response to HPA-1a peptide. Two of these clones were established in long-term culture. Both recognize synthetic as well as naturally processed HPA-1a antigen, and the recognition is restricted by the MHC molecule HLA-DRB3*01:01.

HLA DRB3*01:01 is present in >90 % of the immunized women versus 27 % in the general population (Ahlen et al. 2009). This means that also some other unidentified factors must be crucial for understanding the FNAIT pathophysiology and its prevention (Ahlen et al. 2009). L’Abbe et al. (1992) have found that both HLADRB3*01:01 and HLA DQB*02:01 are associated with alloimmunization to HPA-1a. It has recently been suggested that the presence of two HLA alleles (DRB3*01:01 and DRB4*01:01) in the mother increases the risk and severity of FNAIT and reduces the success of preventive IgG treatment (Loewenthal et al. 2013).

## Anti-HPA Antibodies: Function, Activity and Effect on the Severity of FNAIT

In those pregnant women with detectable anti-HPA-1a antibodies, the disease severity widely varies (Maslanka et al. 2003; Tiller et al. 2013, 2014; Uhrynowska et al. 1997, 2000). IgG is the only antibody class to cross the placenta and only IgG is involved in the pathogenesis (Kumpel 2012; Kumpel and Manoussaka 2012). Several factors may be responsible: antibody type (subclass), concentration, ability to cross the placenta as well as the glycosylation pattern. The interaction with endothelial cells in the fetus may also be of importance (Yougbaré et al. 2015).

Most alloantibodies to platelets and red cells are IgG1, sometimes IgG3 or a mixture of both (Mawas et al. 1997). Antibodies may bind complement on the opsonized target cells. However, the four subclasses of IgG differ both in the ability to cross the placenta as well as of targeted cell destruction. IgG3 has very strong effector functions, but rather poorly transports across the placenta. It has recently been observed that placental transport of this subclass strongly depends on a single-amino-acid polymorphism at position 435; it was found that some allotypes of IgG3 resemble those of IgG1 (Einarsdottir et al. 2014).

IgG’s activity in adherence, phagocytosis and lysis of IgG-coated cells by IgG Fc receptors on macrophages can be modulated by glycosylation. Kapur et al. (2014) reported that platelet alloantibodies in pregnant women lack IgG1-Fc fucosylation. Antibodies with a low amount of fucose displayed higher binding affinity to FcγRIIIa and FcγRIIIb, but not to FcγRIIa, as compared to antibodies with a high amount of Fc fucose. The authors also confirmed that a lowered anti-HPA-1a Fc fucosylation correlates with decreased neonatal platelet counts and increased disease severity in FNAIT patients. Interestingly, in contrast to the FNAIT patients, no changes in core fucosylation were observed for anti-HLA antibodies in refractory thrombocytopenia (post platelet transfusion) which indicates that the level of fucosylation may be antigen dependent and/or related to the immune milieu defined by pregnancy.

It was also found that HPA-1 a alloantibodies are heterogeneous in their ability to interfere with fibrinogen binding to the GPIIbIIIa receptor which may contribute to pronounced bleeding in patients with alloimmune thrombocytopenia (Kroll et al. 2005). For FNAIT pathogenicity, it is also noteworthy that HPA-1a alloantibodies bind the β3 integrin not only on platelets, but also on the surface of vascular endothelial cells via the αVβ3 complex and have a direct effect on endothelial cell spreading and monolayer integrity (van Gils et al. 2009). This may contribute to the increased tendency for FNAIT children to bleed (van Gils et al. 2009). Endothelial cell damage may explain the difficulty in predicting adverse fetal hemorrhagic outcomes from platelet counts alone. Endothelial cell activation and subsequent damage may contribute to explaining FNAIT’s propensity for intraparenchymal ICH, as opposed to the intraventricular hemorrhage more commonly observed in prematurity and other conditions (Althaus et al. 2005; Leeksma et al. 1987).

It is still debatable whether the maternal anti-HPA-1a antibody level during pregnancy is a good predictive factor of disease severity. A correlation between antibody levels in the last trimester and severity of thrombocytopenia was reported by Williamson et al. (1998). Several authors confirmed this observation through measuring the antibody concentration by the monoclonal antibody-specific immobilization of platelet antigens technique (Bertrand et al. 2011; Bertrand and Kaplan 2014; Bertrand et al. 2006; Killie et al. 2008). Antibody concentrations for identifying cases at risk of FNAIT varied from 3 to 28 IU/ml. The positive predictive value of antibody concentration is reported to be similar to the predictive value of obstetric history, which, however, cannot be used for analysis of the first pregnancy. It is suggested that antibody concentration is superior to obstetric history as predictive factor since it has a much higher negative predictive value (Bertrand et al. 2006; Killie et al. 2008). Nonetheless, it is also worthy to note that some studies did not confirm correlation between antibody concentration and thrombocytopenia of the newborn (Bessos et al. 2005; Ghevaert et al. 2007).

## Influence of Other Factors on FNAIT Risk

A correlation has recently been reported between maternal ABO phenotype and ABO genotype with the severity of thrombocytopenia in the newborn; blood group O immunized women were at lower risk of having a child with severe FNAIT than blood group A women. 20 % of the former gave birth to children with severe FNAIT as compared to 47 % of the latter (Ahlen et al. 2012).

The role of anti-HLA antibodies in neonatal thrombocytopenia should also be considered. It is known that in general fetal/maternal mismatch of human leukocyte antigen (HLA) is beneficial for fetal growth and survival (Rugeles and Shearer 2004). HLA antibodies are usually stimulated by pregnancy in healthy women, and appear to have no harmful effect if they are IgM, but it is speculated that FNAIT can occur due to IgG HLA class I antibodies. Anti-HLA antibodies are usually adsorbed rapidly onto the surfaces of fetal cells. It has been confirmed that they can cause complement-mediated platelet aggregation, and it cannot be excluded that they may exacerbate the clinical symptoms in FNAIT. However, the role of concurrent HLA alloantibodies in neonatal thrombocytopenia is still controversial (De Tar et al. 2002; Loewenthal et al. 2013; Salomon et al. 2011; Taaning 2000).

Li et al. (2013) observed that the effect of lipopolysaccharide and Poly I:C stimulation enhanced the immune response to β3 integrin, suggesting that bacterial and viral infections facilitate the risk of fetal disease.

## Prevalence of FNAIT

### Prospective Studies on Thrombocytopenia Due to Alloimmunization

Kamphuis et al. (2014) recently performed a metanalysis of prospective studies available on Medline, Embase and Cochrane database dedicated to FNAIT diagnosis in a general, non-selected newborn population with sufficient access to information on platelet count at birth, bleeding complications and treatment. In the group of six papers, which met the inclusion criteria, one was from Poland and included the largest group of newborns (26,275) (Uhrynowska et al. 2000). In total, Kamphuis’s analysis was based on data from 59,425 newborns and in 89 cases (0.15 %) severe thrombocytopenia was defined by a platelet count <50 × 10^9^/L. Of these, FNAIT was diagnosed in 24 (27 %). In 6 (25 %), ICHs were reported, most likely of antenatal origin. The study confirms that FNAIT is one of the major causes of neonatal thrombocytopenia. Intracranial hemorrhage due to FNAIT occurred in 10 per 10,000 neonates, typically before birth.

### Screening for HPA-1a as a Method for Identifying the Most Frequent and Severe FNAIT

The frequency and clinical presentation of FNAIT was also analyzed in several prospective studies where the screening procedure was similar as for RhD examination. They identified HPA-1a negative women as a risk group for the most frequent and most severe FNAIT. The largest study on FNAIT due to anti-HPA1a included >100,000 pregnancies and was performed in Norway (Kjeldsen-Kragh et al. 2007). The prevalence of at-risk pregnant women with the HPA-1bb genotype was 2.1 % (1990 women) and 210 women (10.6 %) of these were immunized against HPA-1a. In 39 cases, anti-HPA 1a was first-time detected in a sample obtained approximately 6 weeks postpartum. Out of 161 HPA 1a-positive children, 55 had severe thrombocytopenia (<50 × 10^9^/L) including two with ICH. This means that FNAIT occurred in about one in 2000 pregnancies. In Poland during 1999–2003, the HPA-1 phenotype was determined by ELISA in 8031 pregnant women, followed by anti-HPA-1a analysis in HPA-1bb women. In accordance with similar studies, FNAIT was diagnosed for one in 2000 fetuses and newborns (Maslanka et al. 2003).

The systematic review of prospective studies results for HPA-1a alloimmunisation in low risk pregnant women was done by Kamphuis et al. (2010). They summarized the data from 176,084 cases published in ten papers in the period 1985–2007, including two papers described above (Kjeldsen-Kragh et al. 2007; Maslanka et al. 2003). The rates of HPA-1a negative women varied from 1.3–3.1 % of whom 9.7 % developed anti-HPA-1a antibodies of which 31 % had severe FNAIT.

## Clinical Presentation of FNAIT

The clinical presentation of FNAIT varies from asymptomatic thrombocytopenia to a very severe, sometimes lethal ICH (Husebekk et al. 2008; Sachs 2013; Salomon and Rosenberg 2013; Tiller et al. 2014; Uhrynowska et al. 2000). Other consequences of FNAIT have also been described and a recent study has shown that the anti-HPA-1a antibodies or thrombocytopenia may be the reason for decreased birth weight and a too low weight for gestational age which confers a health risk later in life (Tiller et al. 2012b).

Various studies have demonstrated ICH, the most severe clinical symptom of FNAIT, in 10–26 % of mothers with anti-HPA-1a antibodies. Contrary to the other factors causing ICH, in FNAIT intraparenchymal hemorrhage in the temporal lobe is most frequently observed (Dale and Coleman 2002). Fatal outcomes are reported in one-third of cases and various neurodevelopmental consequences may occur in alive children (Kjeldsen-Kragh et al. 2008; Mueller-Eckhardt et al. 1989; Spencer and Burrows 2001).

Clinical presentation data on FNAIT, including the frequency and severity of ICH, are based on the different ways of collecting data and conducting follow up (i.e., prospective, retrospective, observational). Prospective studies on NAIT in nonselected newborns with thrombocytopenia, as those summarized by Kamphuis et al. (2014) (see above), give the best possible incidence estimates of NAIT and NAIT-related ICH. When comparing these studies, it is clearly shown that ICH due to FNAIT in non-screened population is much higher (24/59,425) than that found in the group of women who were antenatally screened for FNAIT risk (HPA-1a determination and antibody detection in HPA-1a negatives) (7/176,084 cases). In all such programs, various antenatal and intrapartum treatment methods are introduced which had apparently reduced the mortality and morbidity associated with FNAIT.

We must also underline the role of observational retrospective study focused on children with FNAIT pregnancies. Such a multicenter study was performed by Tiller et al. (2013) on almost 600 FNAIT and was focused on ICH. The study found that the majority of bleeding occurred by the end of the second trimester and were in most cases devastating. This is at odds with other studies which have suggested that the onset of bleeding is in the third trimester (Spencer and Burrows 2001). Tiller et al. (2013) also found that most cases of ICH occurred in boys and that bleeding is often lethal if the fetus is male. They also found that bleeding often affects the first-born child (Tiller et al. 2013). High levels of maternal alloantibodies are reported as being predictors for low fetal platelet count (Killie et al. 2008). But there is no predictive factor for ICH. Even a low platelet count is not a good predictor. The most likely reason being that some anti-platelet antibodies cause not only platelet destruction but also effect brain endothelium (Kroll et al. 2005; van Gils et al. 2009).

In most FNAIT cases, the disease presents as petechiae, hematomas, melena, sometimes hemoptysis, retinal bleeding and hematuria (Mueller-Eckhardt et al. 1989; Sachs 2013). Platelet counts may continue to fall for some days after delivery. The duration of untreated thrombocytopenia differs from patient to patient; usually between several days or weeks and occasionally it may persist for longer periods (Peterson et al. 2013).

In ~50 % of babies born by mothers with anti-HPA-1a antibodies the platelet count is normal (Kjeldsen-Kragh et al. 2007). However, it is very important to diagnose symptomless FNAIT since there is a disease risk in the next incompatible pregnancy. Such cases must be monitored for anti-HPA1a antibody levels during subsequent pregnancy. The antibody test should also be performed after delivery (~6 weeks) since most HPA-1a negative women who delivered HPA-1a positive babies are immunized at delivery (Kjeldsen-Kragh et al. 2007; Uhrynowska et al. 2000).

## Current Treatment Methods

Several peer reviewed papers on FNAIT management have been published, but there is still no international consensus on how to prevent FNAIT-related sequels (Kanhai et al. 2007; Murphy and Bussel 2007; Rayment et al. 2011; van den Akker and Oepkes 2008; van den Akker et al. 2007). Various strategies are employed when FNAIT is accidentally discovered in a neonate after delivery or when FNAIT is suspected because the woman had previously given birth to an affected child. For obvious reasons, there is no universally accepted strategy for women in their first pregnancy (primigravidae) with anti-HPA-1a antibodies detected within the screening program (Table 1).

**Table 1:** FNAIT current treatment methods

	Clinical situation	Treatment strategy	References
1	FNAIT accidentally discovered in a neonate after delivery: postnatal treatment Subsequent pregnancy must be treated according to strategy 2b or 2c	Depends on the platelet count and clinical symptoms In case of very low platelet count with or without ICH, the newborn should immediately receive a platelet transfusion:	Bussel et al. (2010), Kanhai et al. (2007), Murphy and Bussel (2007), van den Akker et al. (2007)
		Compatible platelets from blood donor or maternal platelets (remove plasma which contain alloantibodies)	
		If antigen negative platelets are not available, the newborn should be transfused with random buffy coat platelets	
		IVIG may also be applied	
2	FNAIT suspected based on patient’s history: the woman has previously given birth to an affected child		
	Prenatal treatment of current and subsequent pregnancy:		
2a		Invasive strategy: FBS and intrauterine transfusion (IUT) of mother platelets once a week (not recommended in many countries)	Overton et al. (2002), van Kamp et al. (2005)
2b		Combined strategy:	Bussel et al. (2010)
		Treatment of the mother with IVIG with or without steroids	
		Diagnostic FBS to evaluate fetal platelet count, combined with preventive IUT with maternal platelets (not recommended in many countries)	
		The mode of delivery depends on fetal platelet count	
2c		Non-invasive strategy:	Kamphuis and Oepkes (2011)
		Treatment of the mother with IVIG with or without steroids, without diagnostic FBS	
		In some countries this line of treatment is followed by spontaneous delivery or by cesarean section a few weeks before term with compatible platelets available for the baby after delivery	
3	FNAIT suspected based on detection of anti-HPA-1a antibodies within the screening program: women in first pregnancy (primigravidae), with no obstetric history		
	Prenatal treatment of current and subsequent pregnancy:		
3a		Conservative management:	Kjeldsen-Kragh et al. (2007)
		No treatment during pregnancy	
		Cesarean section a few weeks prior to term with compatible platelets to be transfused immediately in severely thrombocytopenic neonates	
3b		Combined strategy:	PREVFNAIT study
		Diagnostic FBS with preventive intrauterine transfusion of maternal platelets to evaluate fetal platelet count	
		Treating mothers of thrombocytopaenic fetuses with IVIG with or without steroids	
		The mode of delivery depends on fetal platelet count	

Once FNAIT is diagnosed in the neonate, treatment will depend on the platelet count and clinical symptoms. In cases of very low platelet count, with or without ICH diagnosis, the newborn should immediately receive a transfusion of compatible platelets (Bussel et al. 2010; Kanhai et al. 2007; van den Akker et al. 2007). Maternal platelets may be used; however, the plasma, which contains alloantibodies should be removed. If antigen negative platelets are not available, the newborn should be transfused with random buffy coat platelets. The use of compatible platelets is generally preferred because they produce a higher increment and survive longer (Bussel et al. 2010; Kanhai et al. 2007; van den Akker et al. 2007).

Many neonatologists additionally administer intravenous immunoglobulin (IVIG) which is also applied in milder thrombocytopenia cases (Murphy and Bussel 2007).

Prophylactics prior to delivery is possible in those women who had previously given birth to a child with FNAIT or in a setting where risky pregnancies have been identified through a screening program. As most ICH bleeding in fetuses of women with anti-HPA-1a occurred during pregnancy, predominantly in the firstborn (Tiller et al. 2013), treating fetal thrombocytopenia is of crucial importance. The commonly used dose of weekly IVIG is 1 g/kg maternal weight. However, since IVIG is an expensive multi-donor human blood product with dose-related side effects, checks if lower doses are effective have been attempted. In a recently published paper, which summarized an uncompleted trial, it was suggested that pregnancies can be successfully treated with IVIG at 0.5 g/kg (Paridaans et al. 2015).

As opposed to Rh-disease, intrauterine platelet transfusions (IUTs) are not recommended as the first line treatment in FNAIT, mainly due to the short half-life of transfused platelets and high risk of fetal bleeding complications in repeated procedures. IUTs for therapeutic purposes, would involve a significant risk of pregnancy loss, boostering of HPA-1a-immunization and additional alloimmunisation, and should be limited to selected clinical situations. When diagnostic fetal blood sampling (FBS) is performed, transfusion of platelets is administered to prevent prolonged bleeding from the umbilical vein in thrombocytopenic fetuses. However, minimizing the number of fetal sampling procedures is commonly recommended, and in some countries these procedures have been abandoned altogether. The most common treatment regime of the expectant mother is IVIG, with or without steroids supported by data from the patient’s history. In some countries this line of treatment is followed by cesarean section 2–4 weeks before term with compatible platelets available for the baby after delivery.

In Poland, as in other countries, the therapeutic FNAIT algorithm has gradually evolved from being more invasive to mostly non-invasive. Initially, in the 1990s, FBS for assessing fetal platelet count was offered to all pregnant women with anti-platelet antibodies. Platelet transfusions with platelet concentrates obtained from maternal blood were performed to increase the safety of the procedure and to avoid further alloimmunization. Diagnostic FBS in experienced hands and in combination with immediate platelet transfusion did not, however, cause significant risk as compared to the standard procedure performed in otherwise healthy fetuses. Nevertheless, the procedure is now limited to cases that require measuring platelet levels in patients with anti-HPA-1a antibodies in first pregnancies. As mentioned above, fetal thrombocytopenia will affect only a small group and up to date laboratory data (presence of the HLA DRB3*01:01 antigen, level of antibodies) did not sufficiently inform on the fetal status. In mothers of thrombocytopenic fetuses and in all pregnant women with a history of a FNAIT affected child, IVIG is administered and continued until delivery. If the father is heterozygous regarding the HPA-1 antigen, the fetus genotype is examined by noninvasive detection of HPA-1a allele in fetal DNA isolated from maternal plasma (Avent 2014).

## Perspectives of Immunoprophylaxis

Human platelet antigens are expressed on fetal platelets as early as the 16th gestational week (Gruel et al. 1986) and anti-HPA-1a antibodies can be detected in the first pregnancy. Rates of alloimmunization during the first pregnancy are about 25 % (Killie et al. 2008). In a prospective study of antenatal screening for anti-HPA-1a antibodies, Turner et al. (2005) reported that only one in 25 women were in their first pregnancy upon immunization and in the study of Williamson et al. (1998), 8/33 immunized women were primigravidae. This means that FNAIT resembles hemolytic disease of the fetus and newborn and immunization in many cases is related to delivery; the second child being at risk of the disease. This observation was the starting point for the project funded by EU (PROFNAIT, 2012–2018, http://www.profnait.eu), coordinated by the Norwegian company Prophylix Pharma AS (http://www.prophylixpharma.com) which aims at developing an immunoprophylaxis for HPA-1a negative women who deliver an HPA-1a positive child. The prophylactic drug is based on a polyclonal antibody preparation that does not deviate too much from the current anti-D whose effect is well proven. In spite of efforts to develop anti-D prophylaxis based on engineered monoclonal antibodies, there is still no alternative to anti-D purified from immunized individuals. In the future, however, recombinant anti-HPA-1a can also be considered as prevention against HPA-1a alloimmunization; recombinant high-affinity HPA-1a antibody (B2G1Δnab) developed by Ghevaert et al. (2013) efficiently cleared antibody-sensitized platelets. Monoclonal antibodies examined by Tiller et al. (2012a) induced an antibody-mediated immune suppression against allogeneic platelets in mice as efficiently as polyclonal ones.

Another way to prevent alloimmunization towards the HPA-1a antigen is to induce cellular tolerance against it. This procedure is used therapeutically for adapting the immune system to external antigens or allergens—in allergy, asthma and autoimmune diseases (Cavkaytar et al. 2014). Identifying the major T-cell epitope associated with FNAIT and the availability of clonal HPA-1a-specific CD4^+^ T cells are important contributions in the development of such therapeutic strategies (Skogen et al. 2010).

## Perspectives on New Treatment Methods

Several studies indicate novel treatment modalities (Bakchoul et al. 2013; Ghevaert et al. 2013; Mathiesen et al. 2013) most of which used animal models, although some are also human studies (Ghevaert et al. 2013). The principle is to administer a nondestructive IgG antibody sharing the specificity of the pathogenic antibody to the mother which has retained the ability to be transported across the placenta. Notably, at the molecular level, the IgG given must lack the ability to activate complement; it will not bind classical Fcγ receptors (FcγRs) that induce effector functions, whereas binding to FcRn must be preserved to allow the transplacental transport. By competing-out pathogenic anti-HPA-1a antibodies, these humanized monoclonal antibodies which are constructed in this way may be a novel treatment strategy to prevent anti-HPA-1a-mediated platelet destruction in FNAIT. One example is the human recombinant high-affinity HPA-1a antibody-specific scFv (B2) (Ghevaert et al. 2008, 2013). The B2 antibody is modified by changing the Fc portion which blocks the actual antibody from binding and therefore prevents, or at least ameliorates, fetal and neonatal thrombocytopenia in sensitized mothers who would otherwise be affected; the B2 antibody normally interacts with FcRn, allowing transplacental passage.

Bakchoul et al. (2013) conducted an extensive study of SZ21, a murine monoclonal antibody directed against platelet glycoprotein IIIa. This found that SZ21 F(ab’)_2_ fragments might be therapeutically effective for inhibiting or displacing maternal HPA-1a antibodies from the fetal platelet surface and thus prevent clearance from the circulation. Administration of these fragments not only inhibited the binding of HPA-1a antibodies to circulating human platelets which prevented their clearance, but also displaced the bound HPA-1a antibodies from the platelet surface. Bakchoul et al. (2013) presented further studies on SZ21. The antibody was modified by deglycosylation thereby removing complement activation and phagocytosis whilst in vivo transplacental transport of modified (NGM)-SZ21 was not impaired. When injected into pregnant mice, both native-SZ21 and NGM-SZ21 were transported equally into the fetal circulation where NGM-SZ21 prevented PLT destruction induced by maternal anti-HPA-1a antibodies. This suggests that also humanized, deglycosylated anti-HPA-1a monoclonal antibodies may represent a novel treatment strategy for preventing anti-HPA-1a.

Another approach to antibody modification is to produce antibodies with a hinge region deletion (IgG3ΔHinge) (Mathiesen et al. 2013). IgG3ΔHinge is inactive in both complement-dependent cytotoxicity and antibody-dependent cellular cytotoxicity as measured in functional assays. Modified antibodies are efficiently delivered from the maternal to the fetal compartment in ex vivo placenta perfusions experiments.

Chen et al. (2010) demonstrated, using a mouse model, that FcRn is required for transporting all IgG isotypes to the fetus that includes both transport of pathogenic IgG, as well as human IVIG. They found that FNAIT in mice can be prevented by blocking FcRn with the anti-FcRn monoclonal antibody 1G3. This data suggests that FcRn can also be a potential target for therapy (Chen et al. 2010).

## FNAIT in Poland as well as in Other Countries is Underdiagnosed

Many studies stress that FNAIT is highly underdiagnosed (Sachs 2013; Skogen et al. 2009, 2010; Tiller et al. 2009). One such study by Tiller et al. (2009) demonstrated that the disease is diagnosed in only around 15 % of cases in an unscreened population. Moreover, there are some severely thrombocytopenic newborns who do not have any petechiae, and this may be the reason why some FNAIT cases with ICH are not examined for maternal antibodies against platelets and thus remain undiagnosed.

Based on an incidence of one in 2000, the annual numbers of live births (400,000) and stillbirths (1500) in Poland, about 200 cases of FNAIT can be estimated annually. However, only less than 30 cases are diagnosed. Most cases (~90 %) are diagnosed after birth if petechia or ICH occurs in a newborn. Laboratory results on the mother and newborn verify the diagnosis of FNAIT. Hence, every year many FNAIT cases in escape proper diagnosis and treatment. A low diagnostic rate could also reflect an ignorance of the disease in smaller maternity hospitals. The Institute of Hematology and Transfusion Medicine therefore initiated the project PREVFNAIT: “Prevention of alloimmune thrombocytopenia in fetuses and neonates (AIMPN) in Poland”. Testing of HPA-1a antigen in a large group of pregnant women (~30,000) is planned from different regions of Poland. More than 260 collection sites have been set up. Gynecologists were informed about the project via the Regional Consultant for Obstetrics and Gynecology and the general public by means of an extensive media promotional campaign. It is hoped that such public media exposure will raise the level of awareness about the FNAIT. HPA-1a typing is done by phenotyping (FACS analysis) or by genotyping. All HPA-1a negative (HPA-1bb) women are at risk of immunization and will be tested for anti-HPA-1a antibodies at 16–20, 24, 32 and 40 weeks of gestation along with ~6 weeks after delivery. They will be also tested for HLA immunization to determine anti-HLA class I involvement in FNAIT. Fathers will be tested for their “zygosity” in HPA-1. When the father is heterozygous for HPA-1a/b there is a 50 % chance that the fetus will be compatible with the mother (HPA-1bb) and immunization will not occur where preformed HPA-1a antibodies will not react with fetal platelets. In anti-HPA-1a women, non-invasive determination of fetal genotype will be undertaken through analyzing cell free fetal DNA isolated from maternal plasma to establish if the fetus is incompatible with the mother. Women with antibodies will be offered treatment at the 2nd Department of Obstetrics and Gynaecology, Medical Centre of Postgraduate Education.

All alloimmunised pregnant women with anti-HPA-1a antibodies and with fetal/neonatal thrombocytopaenia or ICH in previous pregnancies will be empirically treated with weekly IVIG 1 g/kg starting from 24 to 28 weeks of pregnancy. Decisions in choosing appropriate therapy for alloimmunised women in their first pregnancies will be made based on individual fetal platelet count. All those women will have offered fetal blood sampling between 24–28 weeks to determine the platelet count. Mothers of thrombocytopenic fetuses will be treated with an IVIG dose of 1 g/kg weekly. Fetal blood sampling will be available to all women in the third trimester (34–36 weeks) for assessing the efficacy of the therapy and for planning the delivery mode. Women with profound thrombocytopenia (platelet count less than 50 × 10^3^/mm^3^), diagnosed close to term, will additionally receive steroids (prednisone 1 mg/kg once daily) and delivery will be by cesarean section.

The second project aim is to find biomarkers for predicting the risk of antibody development and thrombocytopenia in the fetus exposed to maternal alloantibodies. Blood samples will be collected at sites listed on the grant website (http://www.konfliktplytkowy.ihit.waw.pl) and delivered to the Institute of Hematology and Transfusion Medicine in Warsaw for further studies no later than 7 days after collection.

## Similarity of FNAIT and HDN: Rationale for Implementing a Screening Program

HDN due to the feto-maternal incompatibility in the D antigen of RhD system has been effectively limited by introduction of immunoprophylaxis. This can be regarded as a major medical achievement in this field and has consequently saved many lives (Reali 2007). Table 2 compares HDN and FNAIT characteristics and strategies used for prevention and treatment.

**Table 2:** Characteristics of HDN and FNAIT; strategies for prevention and treatment

	HDFN/HDN	FNAIT
Pathogenesis	Alloantibodies to erythrocyte antigens	Alloantibodies to platelet antigens
Clinical symptoms	Hemolytic disease Most severe: hydrops fetalis	Petechiae, hematomas, melena, hemoptysis, retinal bleeding or hematuria Most severe: ICH in fetus or newborn
Immunization	>95 % cases during delivery; HDN risk in the next pregnancy	75 % of cases during delivery; 25 % of cases during pregnancy
Most frequent and most severe: others	Anti-D; risk in RhD negative women (15 % in Caucasian population) Anti-Rhc; E; K; others	Anti-HPA-1a; risk in HPA-1a negative women (2 % in Caucasian population) Anti-HPA-5a; other HPAs
Antigen characteristics	Rh proteins only on erythrocytes	HPA-1a present on integrin β3 on platelets and vascular endothelial cells
HLA restriction	Unknown	HLA DRB3*01:01
Frequency	1/1000–2000 (in the era of immunoprophylaxis)	1/1000–2000
Screening methods	RhD phenotyping in all pregnant women; anti-RhD examination 3× during pregnancy plus antibodies to other RBC antigens tested 2× during pregnancy^a^	Not performed
Doppler USG	Effective in detecting fetal anemia; therapeutic intervention can prevent hydrops fetalis	ICH can be detected but therapeutic intervention which minimize its effect are limited
Treatment	Effective; standardized	Effective; individualized
Immunoprophylaxis	Available for anti-D; not available for others	Under development for HPA-1a

*HDFN* hemolytic disease of the fetus and newborn

^a^Total number of tests in Poland 400,000 pregnancies × 5 = 2,000,000

Immunoprophylaxis for FNAIT prevention is still unavailable but its development is ongoing (Kjeldsen-Kragh et al. 2012; Tiller et al. 2012a). Some studies have confirmed that a routine screening program for identifying HPA1-1a negative women is effective for preventing FNAIT since the monitoring of antibody production and USG examination enable treatment for women at risk of fetal disease to be implemented. Many cases of severe neurological complications or intrauterine fetal deaths due to FNAIT may be thus prevented by early diagnosis. The analysis performed by Killie et al. (2007) confirms that such a program is cost effective. Implementation a screening program is currently under debate (Husebekk et al. 2009; Kjeldsen-Kragh et al. 2008; Skogen et al. 2010). Skogen et al. (2010) discuss this issue in relation to the revised WHO screening criteria based on the newly acquired knowledge and conclude that such screening programs should be implemented.
